# Predicting cervical cancer target motion using a multivariate regression model to enable patient selection for adaptive external beam radiotherapy

**DOI:** 10.1016/j.phro.2024.100554

**Published:** 2024-02-15

**Authors:** Lei Wang, Dualta McQuaid, Matthew Blackledge, Helen McNair, Emma Harris, Susan Lalondrelle

**Affiliations:** The Joint Department of Physics at the Institute of Cancer Research and the Royal Marsden NHS Foundation Trust, Sutton, Surrey, UK

**Keywords:** Cervical cancer, Interfraction motion, Adaptive radiotherapy, Image guided radiotherapy, Mathematical modelling

## Abstract

**Background and purpose:**

Interfraction motion during cervical cancer radiotherapy is substantial in some patients, minimal in others. Non-adaptive plans may miss the target and/or unnecessarily irradiate normal tissue. Adaptive radiotherapy leads to superior dose-volume metrics but is resource-intensive. The aim of this study was to predict target motion, enabling patient selection and efficient resource allocation.

**Materials and methods:**

Forty cervical cancer patients had CT with full-bladder (CT-FB) and empty-bladder (CT-EB) at planning, and daily cone-beam CTs (CBCTs). The low-risk clinical target volume (CTV_LR_) was contoured. Mean coverage of the daily CTV_LR_ by the CT-FB CTV_LR_ was calculated for each patient. Eighty-three investigated variables included measures of organ geometry, patient, tumour and treatment characteristics. Models were trained on 29 patients (171 fractions). The Two-CT multivariate model could use all available data. The Single-CT multivariate model excluded data from the CT-EB. A univariate model was trained using the distance moved by the uterine fundus tip between CTs, the only method of patient selection found in published cervix plan-of-the-day studies. Models were tested on 11 patients (68 fractions). Accuracy in predicting mean coverage was reported as mean absolute error (MAE), mean squared error (MSE) and R^2^.

**Results:**

The Two-CT model was based upon rectal volume, dice similarity coefficient between CT-FB and CT-EB CTV_LR_, and uterine thickness. The Single-CT model was based upon rectal volume, uterine thickness and tumour size. Both performed better than the univariate model in predicting mean coverage (MAE 7 %, 7 % and 8 %; MSE 82 %^2^, 65 %^2^, 110 %^2^; R^2^ 0.2, 0.4, −0.1).

**Conclusion:**

Uterocervix motion is complex and multifactorial. We present two multivariate models which predicted motion with reasonable accuracy using pre-treatment information, and outperformed the only published method.

## Highlights

• Motion of the uterine fundus tip was a poor predictor of interfraction motion.
• After testing 83 potential variables, two multivariate models were formed.
• Predictors were rectal and tumour size, uterine thickness and motion at planning.
• “Movers” can be selected for resource-intensive adaptive radiotherapy.

## Graphical abstract

**Figure:**
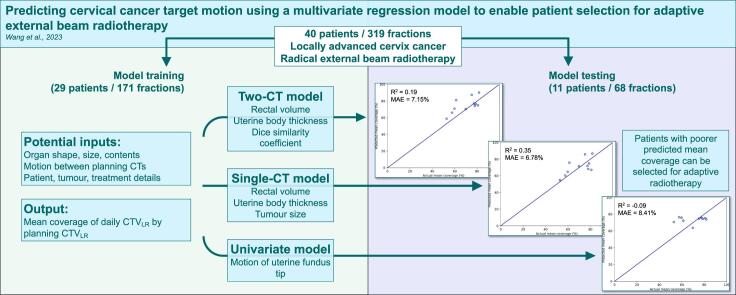

## 1 Introduction

Locally advanced cervical cancer is treated with external beam radiotherapy (EBRT), concurrent chemotherapy and brachytherapy. EBRT is targeted to primary and nodal volumes [1]. Of these, the low-risk clinical target volume (CTV_LR_) contains the uterus, cervix, tumour, parametria and upper vagina. Interfraction motion is individual and can be extensive. The uterine tip as been shown to move as much as 48 mm anteroposteriorly and 32 mm superoinferiorly, primarily related to variation in bladder volume, and the cervix 19 mm anteroposteriorly, primarily related to rectal volume [2]. The challenge is ensuring target coverage, while reducing irradiation of normal tissues. Consequently, practice has evolved from applying large population-based margins, to creating individualised internal target volumes (ITVs), to some centers now adopting an adaptive plan-of-the-day (PotD) approach [3]. Both ITV and PotD approaches require two planning CT scans: one with a full bladder (CT-FB) and another with an empty bladder (CT-EB). The ITV aims to cover the anticipated range of CTV_LR_ motion based on these two scans. PotD involves creating a set of PTVs covering sub-divisions of this range of motion, each smaller than the non-adaptive ITV would have been, and using cone-beam CT (CBCT) to select the appropriate PTV (and thus plan) of the day. Fully online adaptive radiotherapy involves re-segmentation and re-planning while the patient is set up for treatment, and there is emerging technology to achieve this for cervical cancer [4], [5]. PotD and daily online replanning have led to improved dose-volume metrics compared to non-adaptive approaches in cervical cancer [3] and other tumour types [6], [7].

Whichever approach is taken, adaptive radiotherapy is resource-intensive [8]. The benefit is greater for patients with larger degrees of interfraction motion, in whom a non-adaptive ITV will be large and/or fail to cover the motion [9]. Some centers classify patients as “movers” if the tip of the uterine fundus moves more than an arbitrary threshold between CT-FB and CT-EB [10], [11], [12], [13]; movers are selected for PotD while non-movers are treated non-adaptively. This method remains largely unvalidated, though it is observed that some non-movers at planning become movers during treatment [14]. Other centers note, in patients treated with an individualised ITV, that the uterocervix sometimes moves outside the ITV during treatment [15]. Conversely, some patients display less motion at treatment than at planning; the same plan is consistently chosen despite preparation of multiple plans, or the single ITV is unnecessarily large. Consequently, current methods of predicting interfraction target motion during EBRT for locally advanced cervical cancer are prone to under- as well as over-estimating target coverage.

The aim of this study was to create a model from pre-treatment data which could predict the magnitude of individual patients’ CTV_LR_ interfraction motion. The accuracy of this prediction must be great enough to be clinically useful for selecting patients for adaptive radiotherapy.

## 2 Materials and methods

### 2.1 Data collection

We identified 40 patients who had received radical radiotherapy for cervical cancer at our center. All patients had consented to use of their anonymised data for research purposes. Following the EMBRACE-II protocol, all patients had 25 fractions (5 weeks) of external beam radiotherapy followed by high dose rate brachytherapy. Platinum chemotherapy was given alongside external beam radiotherapy in 36 out of 40 patients. Patients were treated supine with a full bladder. At pre-treatment, a CT scan was taken shortly after emptying their bladder (CT-EB), and another one hour after drinking 350 ml water (CT-FB). An MRI was taken in treatment position 30 min after emptying their bladder and drinking 350 ml again, aiming for a half-full bladder. Patients were encouraged to empty their bowels, but no routine bowel preparation was given. Daily CBCTs were rigidly registered to the CT-FB with reference to the pelvic bones. Using RayStation 12-R planning software (RaySearch Laboratories AB, Stockholm, Sweden), the CTV_LR_ was contoured according to the EMBRACE-II protocol [1] on the CT-FB, CT-EB, and a selection of 5–11 CBCTs which represented a wide range of bladder volumes, rectal volumes and uterocervix positions for each patient. Initially, five fractions were selected for each patient using a random number generator; this could be re-rolled up to three times per patient to achieve a roughly even distribution of fractions throughout the duration of treatment. After the five selected fractions were contoured, all CBCTs were visually inspected and further CBCTs contoured if the CTV_LR_ was outside the range of those already contoured, or if the bladder or rectum were subjectively judged to be markedly different in size to the fractions already contoured. In total, 319 scans were contoured.

### 2.2 Selection of variables for testing

A list was compiled of pre-treatment information which could feasibly affect interfraction motion. This included the size of the rectum and/or sigmoid at various superoinferior levels, the contents of the rectosigmoid as reflected by the intensity in Hounsfield units, the size and geometry of the bladder, the volume and geometry of the CTV_LR_ and CTV_HR_, and measures of CTV_LR_ motion between CT-FB and CT-EB. The patient separation, abdominal fat and intra-abdominal space were measured. Patient, tumour and treatment characteristics were collected, including age, menopausal status, body mass index, tumour stage, tumour size on diagnostic MRI, histology and use of concurrent chemotherapy. Some of these variables were hypothesised to affect the fixity of the tumour, while others may influence the physiological mobility of the uterus or the rate of tumour shrinkage during radiotherapy. Where there was uncertainty about the best method for making a measurement, it was done in multiple ways. For example, it is not known whether rectal volume is more or less influential on interfraction motion than the anteroposterior diameter of the rectum, and at which superoinferior level to make the measurement, so each of these permutations were tested. To measure mobility between planning scans, a variety of contour similarity metrics were used, along with distances moved by specific anatomical points such as the tip of the uterine fundus, posteroinferior cervix and center of mass. Thus 83 potential variables were compiled, which can be found in Supplementary Materials A along with details of measurement and rationale.

### 2.3 Interfraction motion

The metric used to describe magnitude of interfraction motion was the percentage coverage of the daily CTV_LR_ contour by the CT-FB CTV_LR_ contour. Coverage was calculated using a custom script within RayStation, and the mean value determined for each patient.

### 2.4 Model development

The data was split into a training set (29 patients, 171 fractions) and test set (11 patients, 68 fractions). The test set was removed prior to model development and only used to evaluate performance. Data were analysed using Python 3.10 in PyCharm CE. The package used for linear regression modelling was scikit-learn version 1.2.

#### 2.4.1 Two-CT model

First, the ability of each baseline feature to predict mean coverage was determined using univariate linear regression. The data were visualised in each case to check whether a mathematical transformation would be appropriate; if so, transformations were applied before further model development. In cases where data transformation improved the R^2^ value but, upon visualisation, the transformed data were felt to be skewed and over-influenced by outliers, the transformation was not applied. All data were normalised. Within each category (see Supplementary Materials A), variables with the greatest R^2^ values were selected and combined in a multivariate linear regression model. Pairwise correlations were calculated between each selected variable to exclude strong inter-correlations. Within the multivariate model, the variable with the highest p-value was removed and the model retrained; this process was repeated until all remaining variables had p-values below 0.15 and were feasible determinants of uterocervix motion. Model coefficients were presented along with their standard error and 95 % confidence interval.

#### 2.4.2 Single-CT model

Using the same steps, an alternative model was developed omitting any variables requiring data from the CT-EB, such as the measures of CTV_LR_ motion and organ geometry on CT-EB. Some centers routinely use a single CT-FB for planning. If motion can be predicted using only one planning scan, some patients may be spared from needing a second CT.

#### 2.4.3 Univariate model

A univariate linear regression was created using the Euclidean distance moved by the tip of the uterine fundus between CT-FB and CT-EB. To our knowledge, this is the only patient selection method mentioned in the literature [10], [11], [12], [13] and routinely used in clinical practice, and remains unvalidated.

Each model was tested on 11 new patients. The models’ performance was reported in terms of R^2^, mean absolute error (MAE) and mean squared error (MSE) of predicted mean coverage compared to true mean coverage. In order to place the models’ performance into a clinical context, we performed a worked example in which a hypothetical radiotherapy center had enough resources to treat a third of their cervical cancer patients with adaptive radiotherapy. A threshold was chosen which would identify a third of patients in the study population. We calculated each model’s accuracy in identifying movers and non-movers on the 11-patient test set.

## 3 Results

Within each category of baseline variables (see Supplementary Materials A), the best-performing on univariate analysis were: Dice similarity coefficient of CTV_LR_ contours on CT-FB and CT-EB (R^2^ = 0.39), tumour size as reported on the diagnostic MRI (R^2^ = 0.30), mean volume of the rectum on CT-FB and CT-EB, divided by the cranio-caudal length of the rectum (R^2^ = 0.26), thickness of the uterine body on CT-FB, measured halfway along and perpendicular to the uterine axis (R^2^ = 0.20), and the superoinferior distance between the top of the bladder and the top of the CTV_LR_ (R^2^ = 0.15). No mathematical transformations were applied.

### 3.1 Two-CT model

**Fig. 1:**
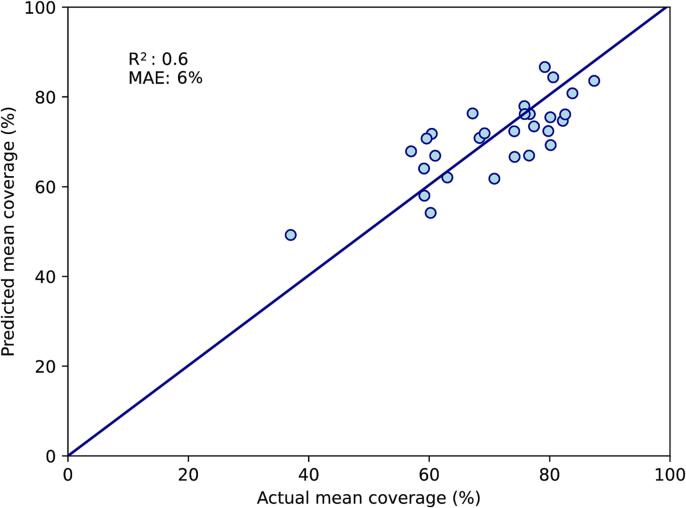

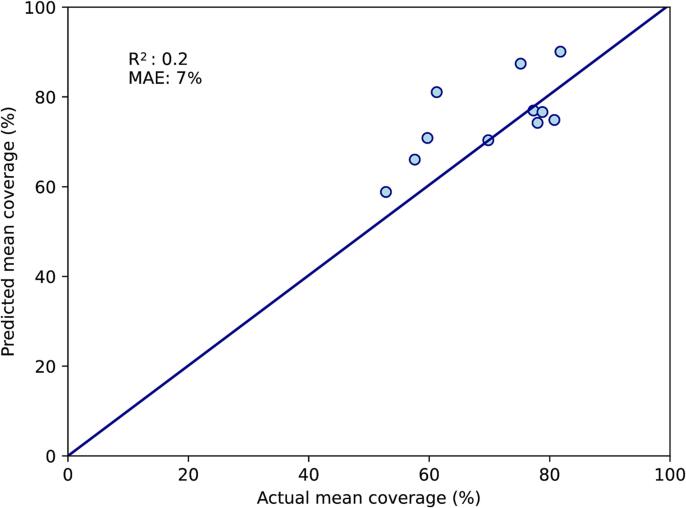
Two-CT multivariate model. A: Performance on the training set (n = 29). B: Performance on the test set (n = 11). This model is based on mean rectal volume at planning, uterine body thickness and dice similarity coefficient at planning. The diagonal line represents perfect predictions. MAE; mean absolute error (%).

When the best-performing variables above were combined in a multivariate model, the variable with the highest p-value was tumour size (p = 0.96). After this was removed, the variable with the highest p-value was the bladder top (p = 0.18) and this was also removed. All three remaining variables (dice similarity coefficient, mean rectal volume and uterine body thickness) had p-value < 0.05, and the F-statistic had p-value < 0.0001. Between these variables, there were no strong inter-correlations with R^2^ above 0.5. On the training set, R^2^ for the correlation between predicted and actual mean coverage was 0.6, with MAE of 6 % (Fig. 1A). On the test set, R^2^ was 0.2 and MAE was 7 % (Fig. 1B).

### 3.2 Single-CT model

**Fig. 2:**
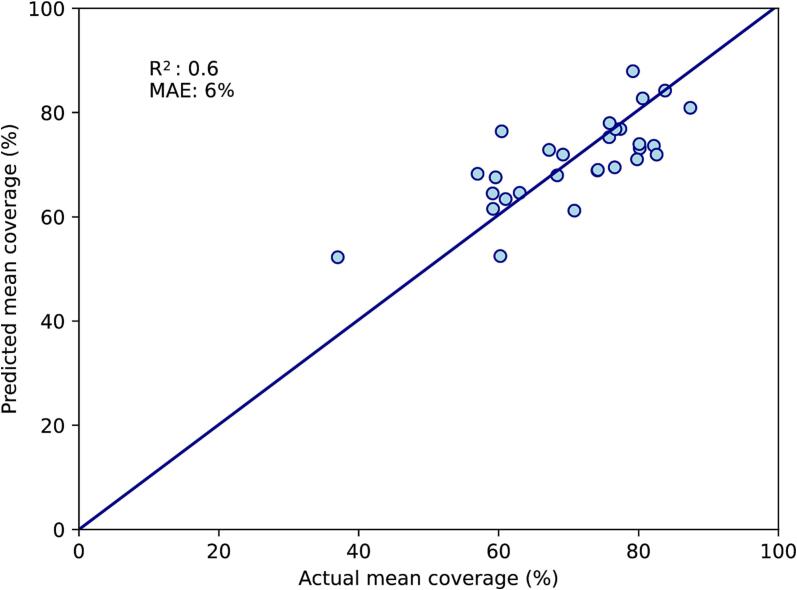

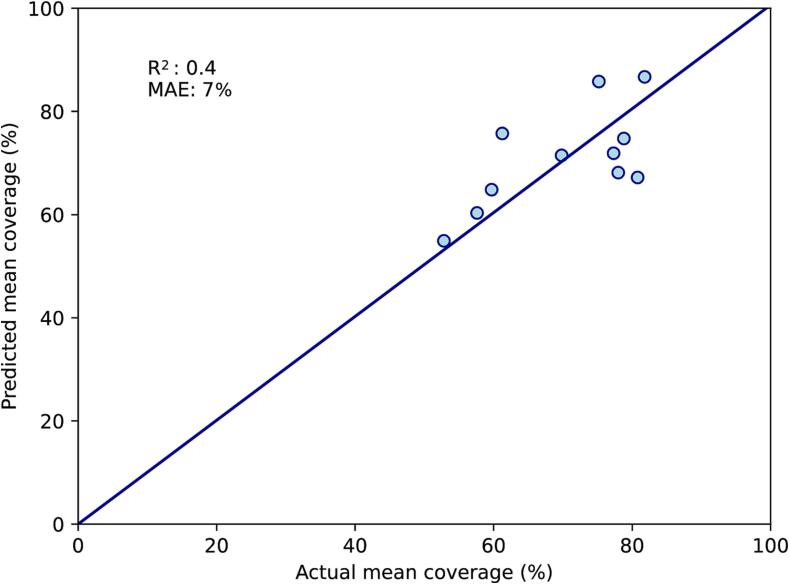
Single-CT multivariate model. A: Performance on the training set (n = 29). B: Performance on the test set (n = 11). This model is based on rectal volume on the full-bladder CT, uterine body thickness and tumour size on diagnostic MRI. The diagonal line represents perfect predictions. MAE; mean absolute error (%).

As mean rectal volume and dice similarity coefficient both required data from the CT-EB, they were removed for the Single-CT model. Mean rectal volume was replaced with rectal volume on CT-FB, which had an R^2^ of 0.24 on univariate linear regression against mean coverage. Within the initial multivariate model, the variable with the highest p-value was the bladder top (p = 0.62). After this was removed, the three remaining variables (rectal volume on CT-FB, uterocervix thickness and tumour size) had p < 0.15, and the F-statistic had p-value < 0.0001. Between these variables, there were no strong inter-correlations with R^2^ above 0.5. On the training set, R^2^ for the correlation between predicted and actual mean coverage was 0.6, with MAE 6 % (Fig. 2A). On the test set, R^2^ was 0.4 and MAE was 7 % (Fig. 2B).

### 3.3 Univariate model

**Fig. 3:**
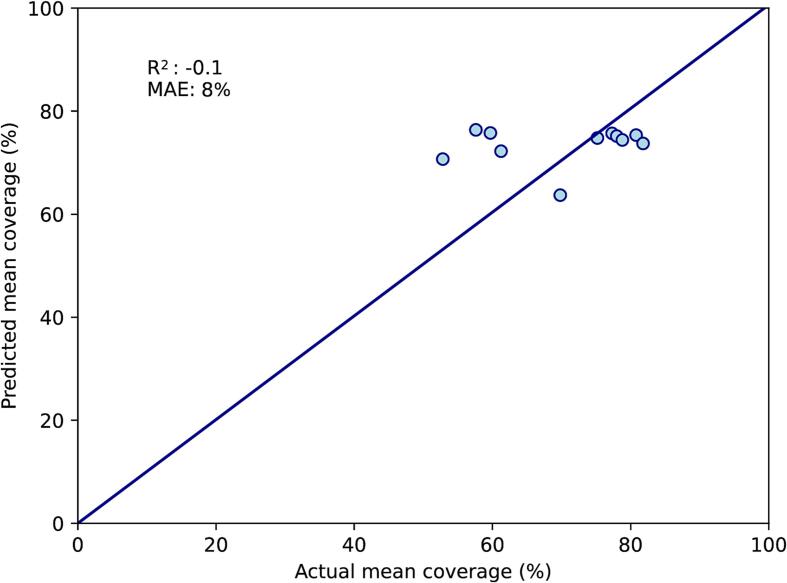

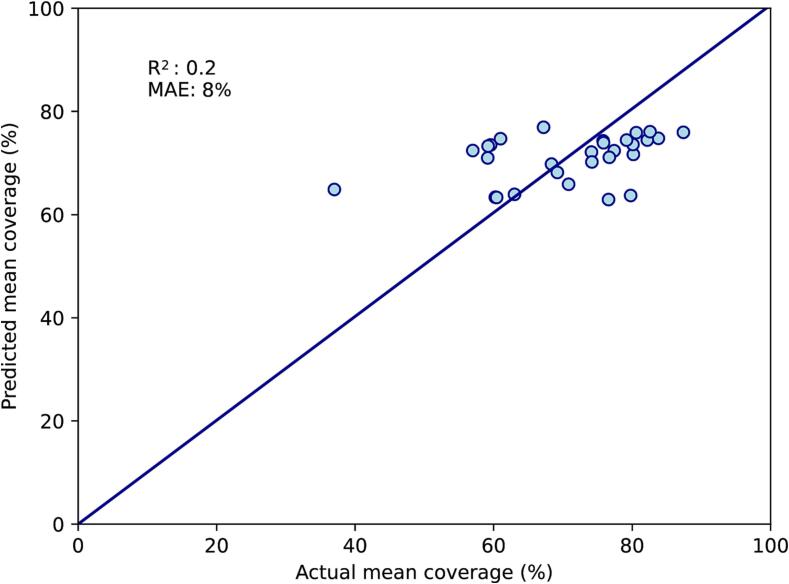
Univariate model. A: Performance on the training set (n = 29). B: Performance on the test set (n = 11). This model is based on the distance moved by the tip of the uterine fundus between CT-FB and CT-EB. The diagonal line represents perfect predictions. MAE; mean absolute error (%).

Using the mean distance moved by the tip of the uterine fundus between CT-FB and CT-EB, a univariate linear regression model was trained. On the training set, R^2^ for the correlation between predicted and actual mean coverage was 0.2, with MAE 8 % (Fig. 3A). On the test set, R^2^ was −0.1 and MAE was 8 % (Fig. 3B).

Model performance metrics are summarised in Table 1. Each model can be replicated using Supplementary Materials B, where we present the coefficients alongside their standard error and 95 % confidence interval. For histograms showing the distribution of key variables in the training and test sets, see Supplementary Materials C.

**Table 1:** Summary of model performance.

	Performance on test set (n = 11)			Performance on training set (n = 29)		
	Mean absolute error (%)	Mean squared error (%^2^)	R-squared	Mean absolute error (%)	Mean squared error (%^2^)	R-squared
Two-CT model	7	82	0.2	6	47	0.6
Single-CT model	7	65	0.4	6	52	0.6
Univariate model	8	110	−0.1	8	99	0.2

### 3.4 Worked example

**Fig. 4:**
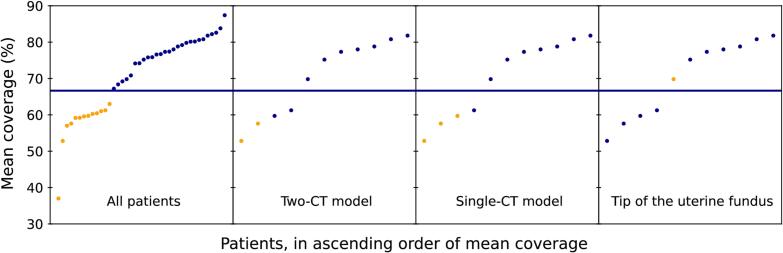
Accuracy of predictions at an example threshold. Blue line; a threshold chosen to select a third of patients for adaptive radiotherapy. Orange dots; patients predicted as movers (patients below the blue line are true movers). Blue dots; patients predicted as non-movers (patients above the blue line are true non-movers). (For interpretation of the references to colour in this figure legend, the reader is referred to the web version of this article.)

A threshold of 66.7 % would identify a third of patients with the worst mean coverage (Fig. 4). On the 11-patient test set, the Two-CT model had an accuracy of 9/11, mis-categorising two movers as non-movers. The Single-CT model had an accuracy of 10/11, mis-categorising one mover as a non-mover. The univariate model had an accuracy of 6/11, mis-categorising all four movers as non-movers, and one non-mover as a mover.

## 4 Discussion

Using three baseline factors, the Two-CT multivariate model trained on 29 patients performed well on 11 new patients, predicting mean coverage with MAE of 7 %. The model over-estimated mean coverage in general. We challenged ourselves to find a model which requires only a single planning CT with a full bladder, which is the practice at some centers. The Single-CT model had similar performance with MAE of 7 %, providing evidence to suggest that some patients could require only a CT-FB; the mean coverage would be predicted using the Single-CT algorithm, after which predicted movers could have a CT-EB and be treated with an individualised ITV or PotD approach, while predicted non-movers could be spared from having a second CT. This approach would need to be validated with further prospective research before routine clinical use.

As expected, uterocervix mobility between CT-FB and CT-EB was correlated with mobility during treatment. Of the various measures of mobility at planning, the dice similarity coefficient between CTV_LR_ contours on CT-FB and CT-EB had the strongest correlation on univariate analysis and was retained as a covariate in the Two-CT multivariate model. Rectal diameter at planning appeared in both multivariate models; the negative coefficient (see Supplementary Materials B) indicates that larger rectal volume at planning was associated with less mean coverage during treatment (more interfraction motion). Uterine body thickness appeared in both multivariate models with a positive coefficient, indicating that a thicker (less physically pliant) uterus was associated with greater mean coverage (less interfraction motion). Tumour size was a covariate in the Single-CT model with a positive coefficient, indicating that a larger, bulker tumour was associated with greater mean coverage (less interfraction motion). These are all commonly observed phenomena in our experience, which supports the biological feasibility and robustness of both multivariate models.

Bladder volume is a notable omission from the list of key variables. During model development, we trialled nine different measures of bladder size and shape (see Supplementary Materials A) but all had very poor correlations with interfraction motion apart from the superoinferior distance between the top of the bladder and the top of the CTV_LR_, which had a weak correlation and was subsequently excluded from both multivariate models. Bladder volume is known to be a key determinant of uterocervix position on any given treatment day [2], but a patient selection model requires bladder volume at *planning* to be predictive of uterocervix motion during *treatment*, for which there is no evidence in the literature.

On univariate linear regressions, R^2^ values were moderate at best (ranging from 0 to 0.39), showing that interfraction motion in the female pelvis cannot be predicted accurately using any single variable. The only published methods of selecting cervical cancer patients for adaptive radiotherapy have been based on motion of the tip of the uterine fundus [10], [11], [12], [13], but on our data it was not a robust predictor of the magnitude of subsequent interfraction motion, with an R^2^ value of −0.1 and larger MAE and MSE than both the multivariate models.

To our knowledge, ours is the first study linking interfraction motion of the cervical cancer CTV_LR_ with multiple patient-specific baseline factors. Motion of the cervical cancer CTV_LR_ can be random and difficult to predict, as the target is a complex shape, and motion is a combination of displacement, deformation and tumour shrinkage over time [16], [17], [18]. This makes online adaptive radiotherapy an attractive prospect. The resource cost has proven a barrier to widespread implementation [19]. It is desirable to select patients with relatively more motion in order to best allocate resources.

Existing studies have tended to measure motion of isolated anatomical points, such as the tip of the uterine fundus [20] or posterior cervix [2], [16], [21]. These methods are prone to missing motion of other parts of the CTV_LR_. Studies which described complex deformation of three-dimensional organ shapes have used deformable image registration and deformation vectors to plot heat maps of inter- and interfraction motion [22]. Attempts have been made to simplify the three-dimensional uterocervix shape such that it can be manipulated mathematically, with varying success [23], [24], [25]. Principal component analysis has been useful in prostate cancer, with only one or two components required to describe the prostate CTV shape [26], but the cervix CTV_LR_ is more complex. For regression modelling, we needed a single metric. Mean coverage was chosen because it is a common metric used in cervical PotD and adaptive radiotherapy studies [13], [27], [28], [29], [30], and target coverage is one of the most clinically relevant concerns in radiotherapy.

All scans were contoured by the same experienced observer. This contributed to the success of the model, as it removed interobserver variability, although intraobserver variability remained. Interobserver variation is greater than intraobserver variation among clinicians and physicists contouring the pelvis on CBCT [31]. However, data collection was resource-intensive. To obtain these 40 datapoints, 319 scans were manually contoured and approximately 1000 scans were visually inspected. Enlarging the dataset would be challenging, though it would undoubtably improve the model accuracy and may identify further key variables. Furthermore, these models have been validated using data from only a single center. Differences in patient positioning and preparation at other centers may affect the degree of interfraction motion and render other variables to be more important. Before widespread use, the models should be validated or refined by data from other centers.

Other studies have demonstrated improvement in various calculated dose-volume metrics when using adaptive radiotherapy in cervical cancer [3], [8]. The resource burden varies depending on which approach is taken, and includes the cost of machines, software and staff training. Treatment time can be lengthened, for example, the mean time taken for a single plan selection was 2.4 min in one study [10] and 15 min in another [12], and using the Ethos system (Varian Medical Systems, CA) there was an average of 29 min between CBCT acquisition and beam-on [32]. There is no established threshold for distinguishing movers from non-movers or for selecting patients for adaptive radiotherapy, as this needs to be a local decision balancing resource availability and anticipated benefit. Without a patient selection method, centers unable to treat *all* patients are unable to treat *any* patients. Our study offers centers the ability to treat a subset of patients based upon anticipated motion. For example, well-resourced centers may choose to treat all cervical cancer patients with adaptive radiotherapy. Other centers may wish to select the half or third of their patients with the greatest predicted motion. Indeed, as non-adaptive centers experiment with adopting adaptive radiotherapy, they may wish to treat only patients with a very high predicted motion (low predicted mean coverage), and gradually lower the threshold over time, treating more patients as the workflow gains fluency.

Once clinically implemented, the model’s performance may be assessed prospectively by auditing the number of predicted movers who had PotD but required only the full-bladder plan (false positives) and the number of predicted non-movers who started non-adaptive treatment but required re-planning (false negatives). In workflows that involve daily online contouring, the model can be evaluated using the coverage of daily contours, and even updated continuously.

In conclusion, using information available at the planning stage, two multivariate models were created which both accurately predicted patients’ mean coverage by the CT-FB CTV_LR_ during subsequent treatment. They key variables were based on rectal volume, dice similarity coefficient at planning, uterine body thickness and tumour size. Patients with relatively more predicted motion (lower mean coverage) may be selected for resource-intensive adaptive strategies. One of these models requires data from only one planning CT, raising the possibility that some patients (predicted non-movers) may be spared from having a second CT. These multivariate models performed better than a univariate model based on the distance moved by the tip of the uterine fundus.

## CRediT authorship contribution statement

**Lei Wang:** Conceptualization, Methodology, Investigation, Validation, Writing – original draft, Writing – review & editing. **Dualta McQuaid:** Methodology. **Matthew Blackledge:** Methodology, Investigation, Validation, Writing – review & editing. **Helen McNair:** Conceptualization, Methodology, Writing – review & editing, Supervision. **Emma Harris:** Conceptualization, Methodology, Funding acquisition, Writing – review & editing, Supervision, Validation. **Susan Lalondrelle:** Conceptualization, Funding acquisition, Methodology, Writing – review & editing, Supervision.

## Declaration of Competing Interest

The authors declare the following financial interests/personal relationships which may be considered as potential competing interests: Lei Wang is part-funded by the National Institute for Health Research (NIHR) Biomedical Research Centre at The Royal Marsden NHS Foundation Trust and The Institute of Cancer Research, London; and part-funded by Elekta Ltd. Helen McNair is funded by a National Institute for Health Research and Health Education England (HEE/NIHR) Senior Clinical Lectureship (ICA-SCL-2018–04-ST2-002). Emma Harris has received research funding from Elekta Ltd and Cancer Research UK Programme Foundation Award A23557. Susan Lalondrelle has received research funding and speaking fees from Elekta Ltd.
