# Supplementary material for: Predicting cervical cancer target motion using a multivariate regression model to enable patient selection for adaptive external beam radiotherapy
Source: Phys Imaging Radiat Oncol. 2024 Feb 15;29:100554. doi: 10.1016/j.phro.2024.100554 (PMC10901141; doi:10.1016/j.phro.2024.100554)

**Supplementary Materials**

**Which cervical cancer patients need adaptive planning? Predicting target motion using a multivariate regression model**

Table of Contents

[A. Full list of independent variables investigated 1](#_Toc151645132)

[Measures of rectal and sigmoid size (21) 1](#_Toc151645133)

[Measures of rectal and sigmoid contents (12) 3](#_Toc151645134)

[Measures of bladder size and shape (9) 3](#_Toc151645135)

[Measures of CTV_LR_ (uterocervix) size and shape (6) 4](#_Toc151645136)

[Measures of CTV_LR_ (uterocervix) mobility between CT-FB and CT-EB (12) 4](#_Toc151645137)

[Other CT measurements (3) 5](#_Toc151645138)

[Patient demographics (11) 5](#_Toc151645139)

[Tumour and treatment characteristics (9) 5](#_Toc151645140)

[B. Model details 6](#_Toc151645141)

[Two-CT model 6](#_Toc151645142)

[Single-CT model 6](#_Toc151645143)

[Univariate model 7](#_Toc151645144)

[C. Distribution of data 7](#_Toc151645145)

## A. Full list of independent variables investigated

All volumes are in cubic centimeters. All lengths and distances are in centimeters.

### Measures of rectal and sigmoid size (21)

- Antero-posterior diameter of the rectum on CT-FB, measured at the axial level of the CTV_LR_ center of mass (“rectal_size_fb”) or the CTV_HR_ center of mass (“rectal_sizec_fb”). It is unclear at which axial level the rectal diameter should be measured. Two anatomically meaningful and reproducible levels are trialled. The level of the pubic symphysis was also considered, but thought to be too low to meaningfully impact motion of the uterocervix.
- Mean antero-posterior diameter of the rectum on CT-FB and CT-EB, measured at the axial level of the CTV_LR_ center of mass (“rectal_size_mean”) or the CTV_HR_ center of mass (“rectal_sizec_mean”).
- Difference in antero-posterior diameter of the rectum between CT-FB and CT-EB, measured at the axial level of the CTV_LR_ center of mass (“rectal_size_diff”) or the CTV_HR_ center of mass (“rectal_sizec_diff”). The hypothesis is that a patient prone to rapid changes in rectal diameter within the space of two planning CTs may be particularly prone to interfractional motion.
- Ratio of the antero-posterior diameter of the rectum between CT-FB and CT-EB, measured at the axial level of the CTV_LR_ center of mass (“rectal_size_ratio”) or the CTV_HR_ center of mass (“rectal_sizec_ratio”).
- Volume of the rectum on CT-FB, divided by the cranio-caudal length of the rectum (“rectal_vol_fb”).
- Mean volume of the rectum on CT-FB and CT-EB, divided by the cranio-caudal length of the rectum (“rectal_vol_mean”).
- Difference in volume of the rectum between CT-FB and CT-EB, divided by the cranio-caudal length of the rectum (“rectal_vol_diff”).
- Ratio of the volume of the rectum between CT-FB and CT-EB, divided by the cranio-caudal length of the rectum (“rectal_vol_ratio”).
- Volume of the sigmoid on CT-FB, divided by the cranio-caudal length of the sigmoid (“sig_vol_fb”).
- Mean volume of the sigmoid on CT-FB and CT-EB, divided by the cranio-caudal length of the sigmoid (“sig_vol_mean”).
- Volume of the rectosigmoid on CT-FB, divided by the cranio-caudal length of the rectosigmoid (“rectosig_vol_fb”).
- Mean volume of the rectosigmoid on CT-FB and CT-EB, divided by the cranio-caudal length of the rectosigmoid (“rectosig_vol_mean”).
- Volume of each cranio-caudal quartile of the rectosigmoid, divided by length (“rectosig1_vol”, “rectosig2_vol”, “rectosig3_vol” and “rectosig4_vol”). The rectogsigmoid was sub-divided to investigate whether a particular section has greatest influence on interfractional motion. All these volumes are on the CT-FB.
- Volume of the 2 cm cranio-caudal section of the rectosigmoid directly posterior to the CTV_LR_ center of mass (“rs2cm_vol”).

### Measures of rectal and sigmoid contents (12)

- Mean intensity in Hounsfield units in the rectum on CT-FB (“rectal_int”). Gas contents have low intensity. Liquid and solid contents have high intensity. The mean intensity reflects the mix of contents in the rectum.
- Mean intensity in Hounsfield units in the sigmoid on CT-FB (“sig_int”).
- Mean intensity in Hounsfield units in the rectosigmoid on CT-FB (“rectosig_int”).
- Mean intensity in Hounsfield units in the 2 cm cranio-caudal section of the rectosigmoid directly posterior to the CTV_LR_ center of mass (“rs2cm_int”).
- Mean intensity in Hounsfield units in the “rectal ball” on CT-FB, a simple surrogate structure consisting of a rollerball at the level of the CTV_LR_ center of mass (“rectalball_int”).
- Percentage gas within the rectum on CT-FB (“rectal_gas”).
- Percentage gas within the sigmoid on CT-FB (“sigmoid_gas”).
- Percentage gas within the rectosigmoid on CT-FB (“rectosig_gas”).
- Percentage gas within each cranio-caudal quartile of the rectosigmoid (“rectosig1_gas”, “rectosig2_gas”, “rectosig3_gas” and “rectosig4_gas”).

### Measures of bladder size and shape (9)

- Bladder width on CT-FB measured at its widest part (“bladder_width_fb”).
- Bladder width on CT-EB measured at its widest part (“bladder_width_eb”).
- Difference between bladder widths on CT-FB and CT-EB (“bladder_width_diff”). The hypothesis is that patients with an inadequate range of bladder volumes at planning may have unexpectedly more interfractional motion during radiotherapy.
- Ratio between bladder widths on CT-FB and CT-EB (“bladder_width_ratio”).
- Bladder volume on CT-FB (“bladder_vol_fb”).
- Bladder volume on CT-EB (“bladder_vol_eb”).
- Difference between bladder volumes on CT-FB and CT-EB (“bladder_vol_diff”).
- Ratio between bladder volumes on CT-FB and CT-EB (“bladder_vol_ratio”).
- Supero-inferior distance between the top of the CTV_LR_ and the top of the bladder (“bladder_top”). This may be a more sensitive measure of sufficient bladder filling.

### Measures of CTV_LR_ (uterocervix) size and shape (6)

- Volume of the CTV_LR_ on CT-FB (“uterocervix_vol_fb”). The hypothesis is that a bulky uterus may be less mobile.
- Mean volume of the CTV_LR_ between CT-FB and CT-EB (“uterocervix_vol_mean”).
- Length of the uterine axis on CT-FB (“axis_len”). The hypothesis is that a long but thin uterus may be more mobile.
- Uterine axis angle, with 0 pointing vertically downwards, 90 pointing horizontally anteriorly, 180 pointing vertically, and 270 pointing horizontally posteriorly (“axis_angle”).
- Thickness of the uterine body on CT-FB (“uterocervix_thickness”).
- Volume of the CTV_HR_ on MRI (“tumour_vol_mri”). The hypothesis is that a bulky tumour may be less mobile.

### Measures of CTV_LR_ (uterocervix) mobility between CT-FB and CT-EB (12)

- Hausdorff distance (“hd_uterocervix”).
- Dice similarity coefficient (“dsc_uterocervix”).
- Minimum surface-to-surface distance (“minssd_uterocervix”).
- Maximum surface-to-surface distance (“maxssd_uterocervix”).
- Mean surface-to-surface distance (“meanssd_uterocervix”).
- Euclidean distance moved by the tip of the uterine fundus (“mvt_U_eucl_eb”), the postero-inferior cervix (“mvt_CA_eucl_eb”), the CTV_LR_ center of mass (“mvt_uCoM_eucl_eb”) and the CTV_HR_ center of mass (“mvt_tCoM_eucl_eb”).
- Antero-posterior distance moved by the most anterior point of CTV_LR_ (“mvt_Ay_eb”).
- Superio-inferior distance moved by the most superior point of CTV_LR_ (“mvt_Sz_eb”).
- Change in the uterine axis angle between CT-FB and CT-EB (“mvt_axis_angle”).

### Other CT measurements (3)

- Patient separation (“pt_sep”).
- Distance between anterior skin surface and abdominal wall (“peripheral_fat”).
- Distance between abdominal wall and posterior skin surface (“central_fat”). The hypothesis is that a larger central abdominal space allows more organ motion.

### Patient demographics (11)

- Age in years (“age”).
- White or other ethnicity (“white” – binary).
- Menopausal status (“postmenopausal” – binary). Changes are known to occur in the uterus after menopause.
- Parity (“parity” – binary). Previous pregnancies may stretch and alter the uterus.
- Previous pelvic surgery (“pelvic_surgery” – binary). The presence of adhesions may limit mobility.
- Presence of uterine fibroids (“fibroid” – binary).
- Height in metres (“height”).
- Weight in kilograms (“weight”).
- Body mass index (“bmi”).
- Smoking status (“smoking” – 2 for current smoker, 1 for ex-smoker, 0 for non-smoker). Smoking is hypothesised to reduce tumour response to radiotherapy due to hypoxia; a tumour may be less mobile if it remains bulky for longer.
- Alcohol (“alcohol” – binary).

### Tumour and treatment characteristics (9)

- Tumour stage (“figo”). More advanced tumours may be more bulky, tethered to surrounding structures and less mobile. This measure may be confouded by nodal staging; positive nodes confer stage III while the primary might not necessarily be advanced.
- Tumour size as reported on the diagnostic MRI (“size”).
- Tumour involvement of the uterus, vagina, parametria or nodes (“uterus”, “vagina”, “parametria” and “nodes” respectively – all binary).
- Tumour grade (“grade”). High grade tumours may shrink more rapidly, resulting in greater changes in target shape towards the end of the radiotherapy course.
- Tumour histology (“squam” – binary).
- Use of concurrent chemotherapy (“ctx” – binary). Concurrent chemotherapy may lead to faster tumour shrinkage.

## B. Model details

### Two-CT model

|  | Coefficient | | | Normalisation | |
| --- | --- | --- | --- | --- | --- |
|  | Value | Standard error | 95% confidence interval | Mean | Standard deviation |
| Mean rectal volume (cubic centimeters) | -4.97 | 1.48 | -8.01, -1.93 | 8.38 | 4.35 |
| Uterine body thickness (centimeters) | 3.76 | 1.60 | 0.46, 7.07 | 3.56 | 1.18 |
| Dice similarity coefficient | 3.81 | 1.65 | 0.42, 7.21 | 0.63 | 0.16 |
| (Intercept) | 70.99 | 1.37 | 68.17, 73.82 |  |  |

### Single-CT model

|  | Coefficient | | | Normalisation | |
| --- | --- | --- | --- | --- | --- |
|  | Value | Standard error | 95% confidence interval | Mean | Standard deviation |
| Full-bladder rectal volume (cubic centimeters) | -5.49 | 1.55 | -8.69, -2.30 | 8.38 | 4.59 |
| Uterine body thickness (centimeters) | 4.59 | 1.71 | 1.08, 8.10 | 3.56 | 1.18 |
| Tumour size (centimeters) | 2.58 | 1.73 | -0.97, 6.14 | 4.97 | 2.3 |
| (Intercept) | 70.99 | 1.44 | 68.03, 73.95 |  |  |

### Univariate model

Predicted mean coverage

= -2.49 × distance moved by the uterine fundus tip (centimeters) + 77.5

## C. Distribution of data

The following histograms show the distribution of values of mean coverage and each of the key variables. Blue bars and lines; training data. Red bars and lines; test set. Dotted line; mean. Dashed line; median.


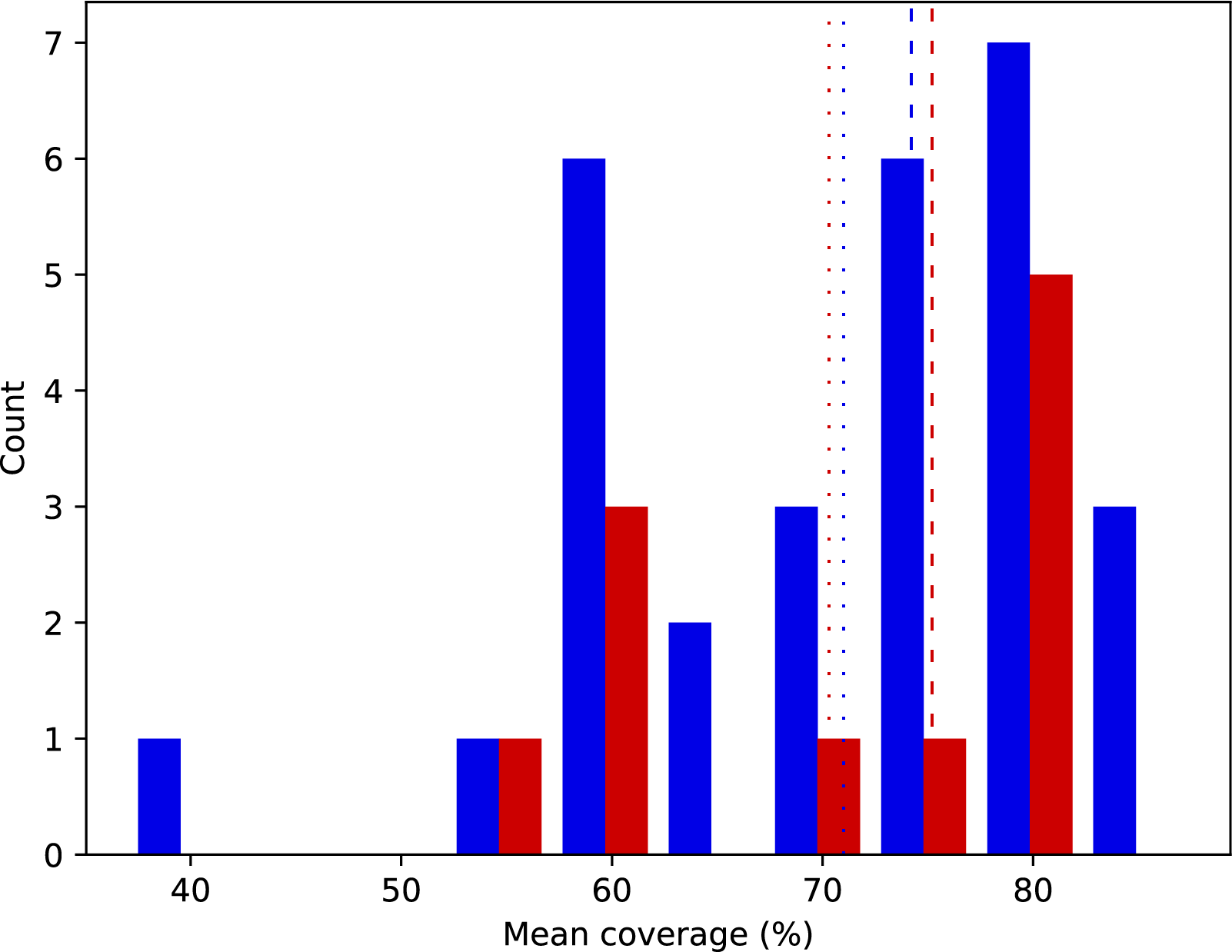


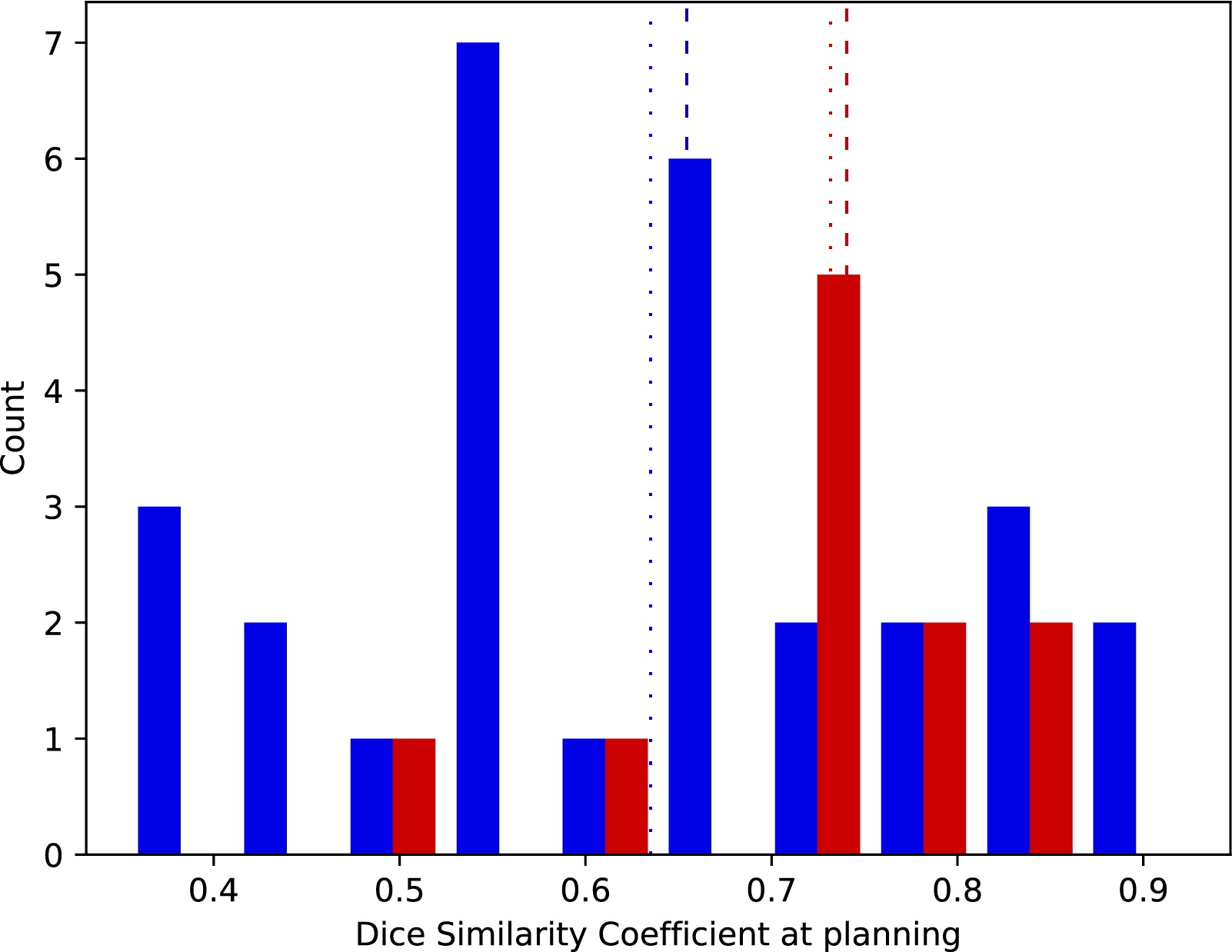


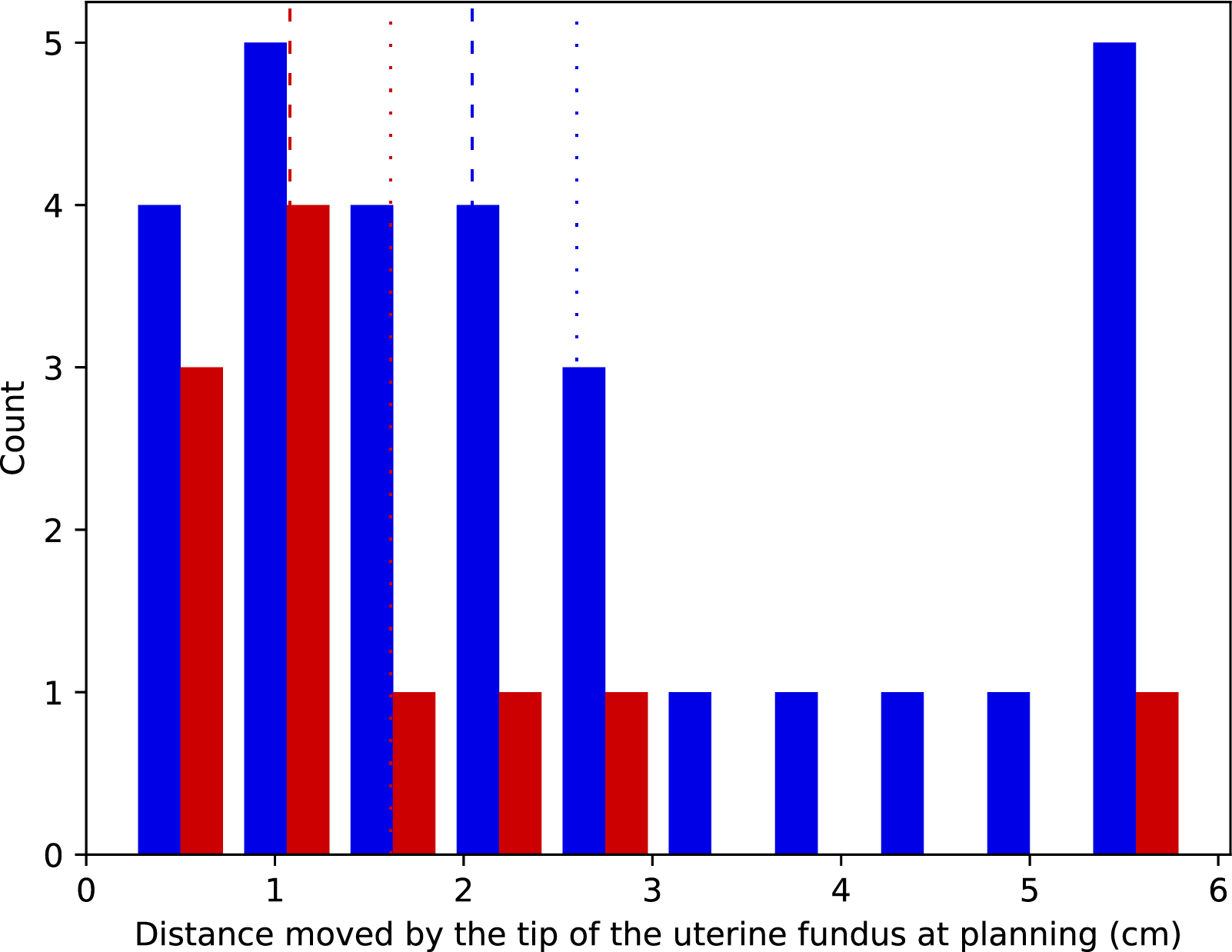


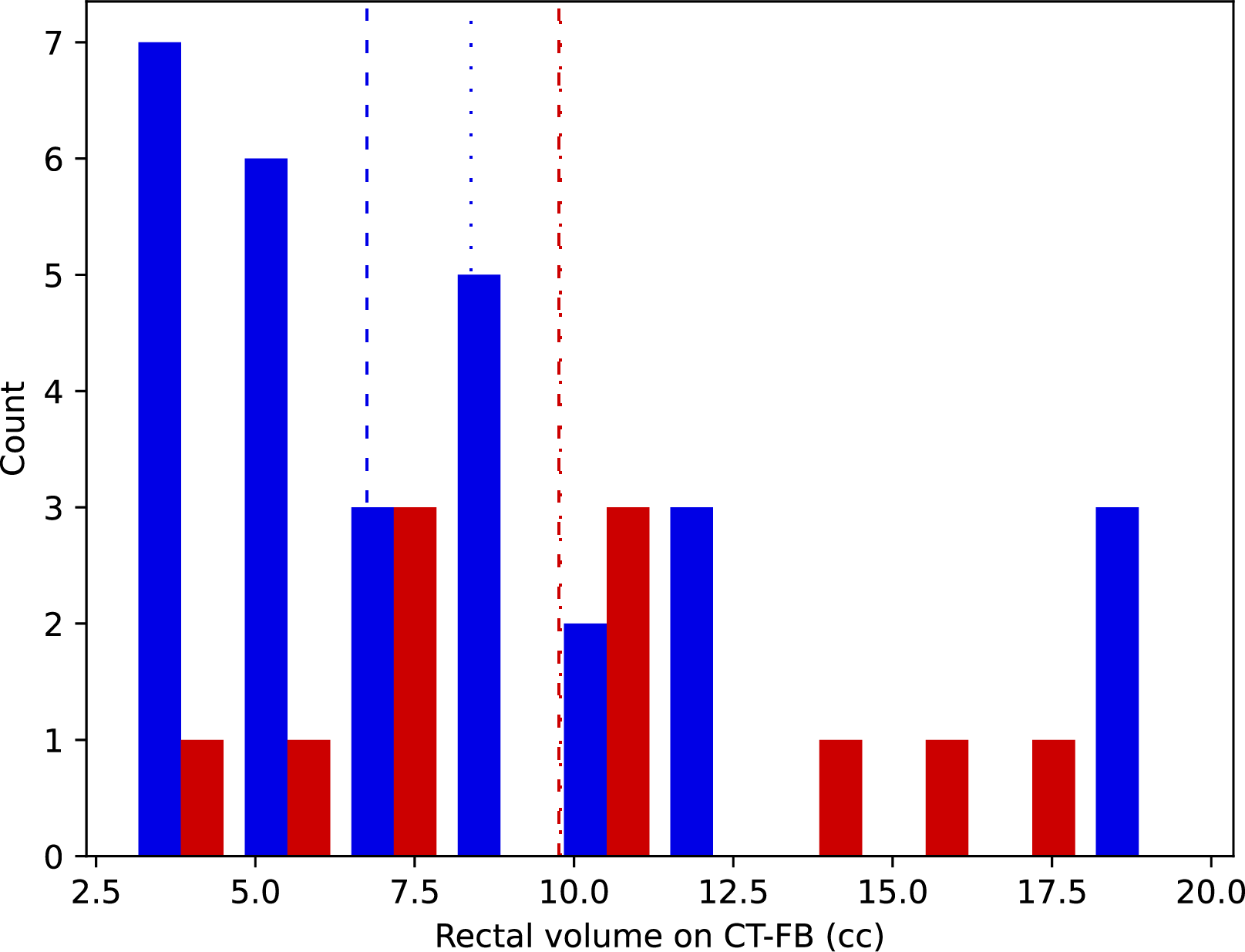


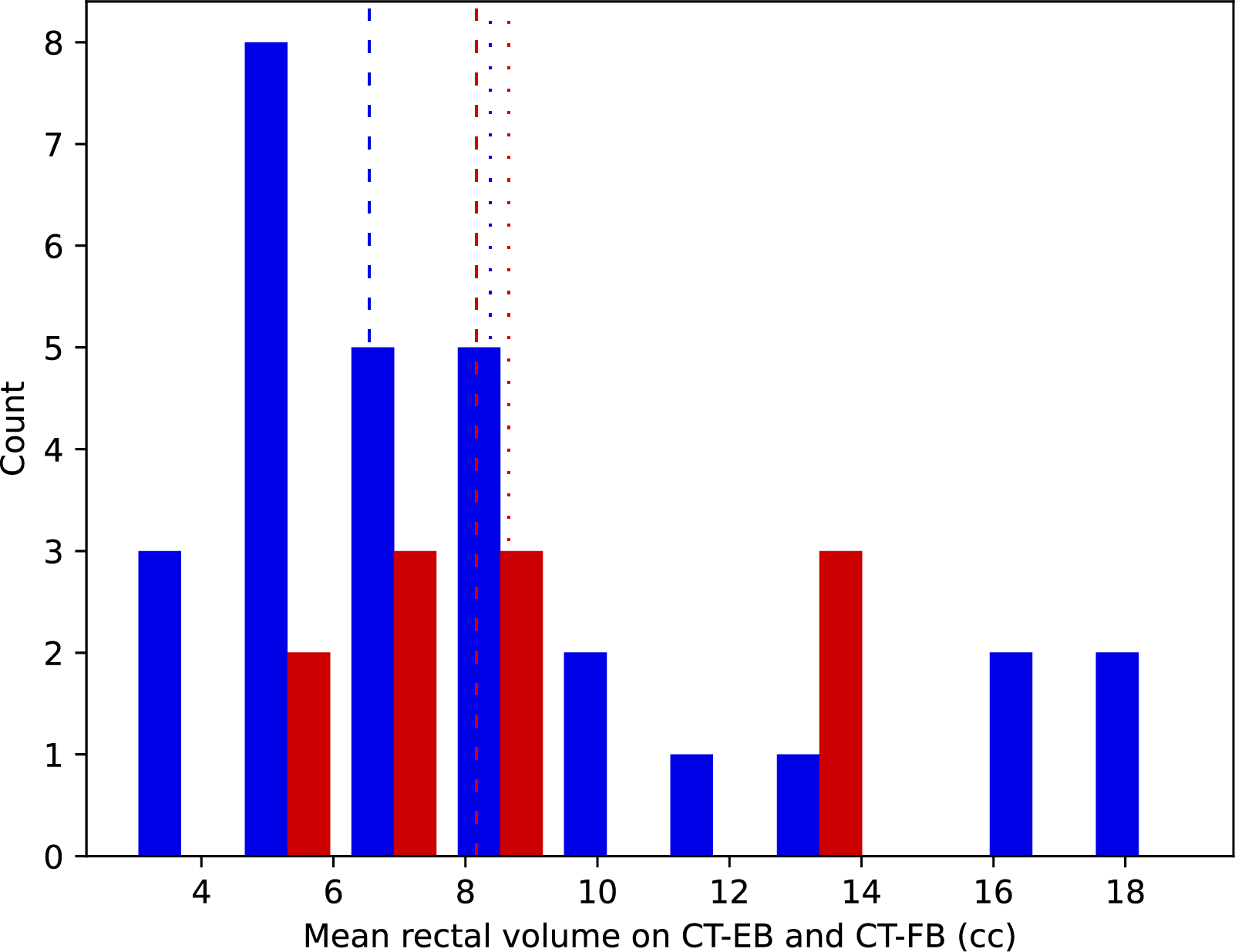


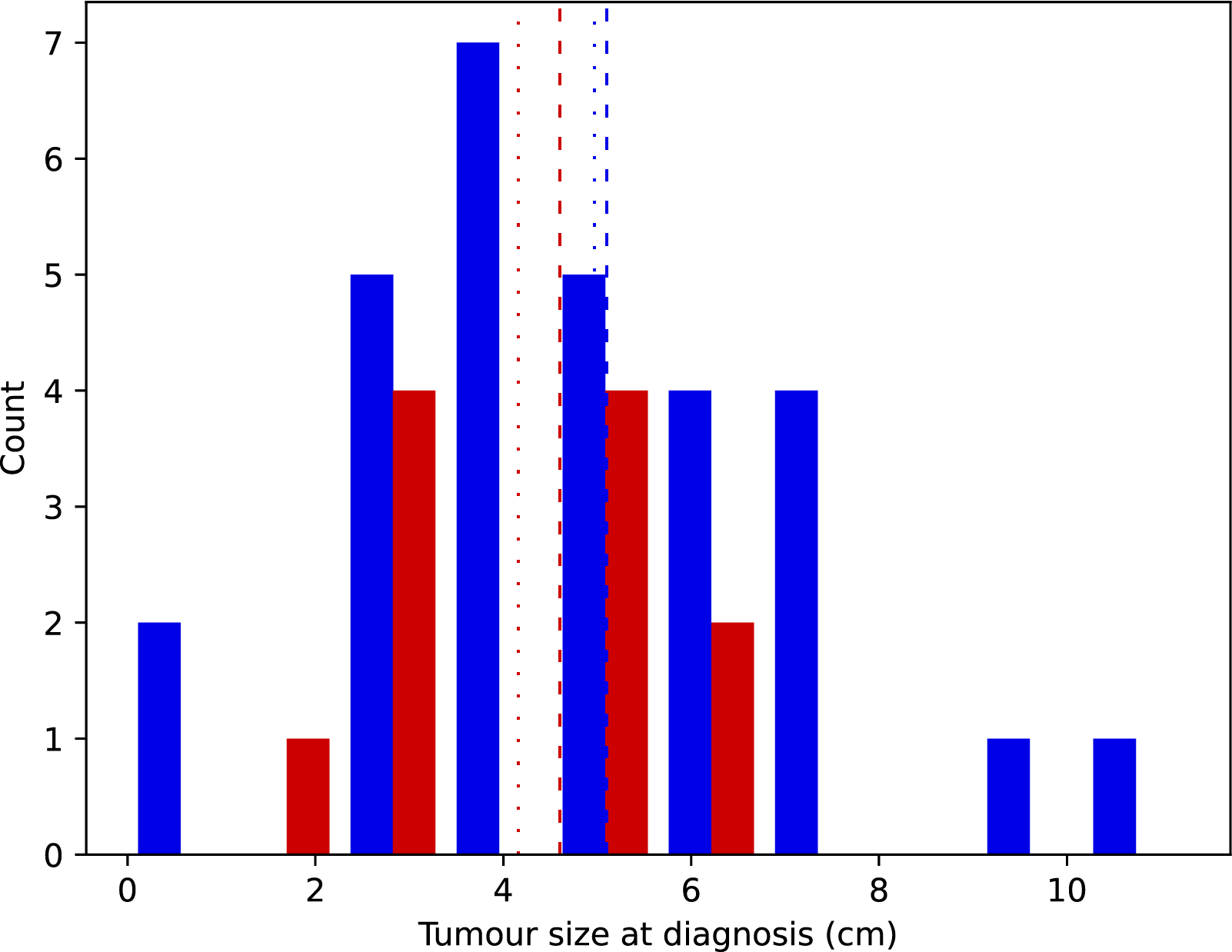


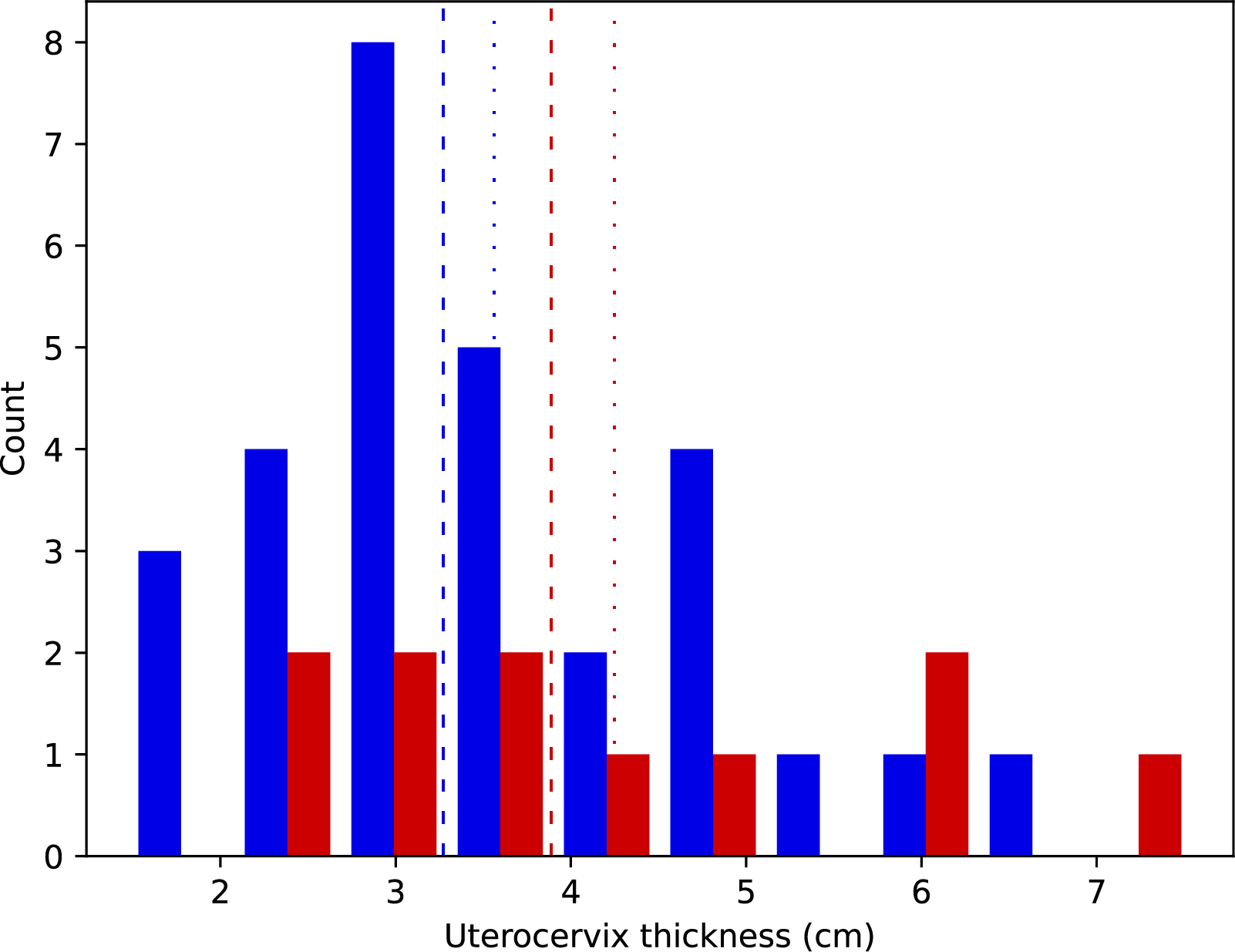

Supplement: Supplementary Data 1 [file mmc1.docx]
